# Beyond Marital Dissolution: Role Transitions and Identity Reconstruction of Divorced Older Women

**DOI:** 10.1093/geroni/igaf122.2493

**Published:** 2025-12-31

**Authors:** Shiau-Fang Chao, Yueh-Tzu Wang, Chien-Chou Hou, Ju-Ping Lin

**Affiliations:** National Taiwan University, Taipei, Taipei, Taiwan (Republic of China); National Taiwan University, Taipei, Taipei, Taiwan (Republic of China); Chung Shan Medical University, Taichung, Taichung, Taiwan (Republic of China); National Taiwan Normal University, Taipei, Taipei, Taiwan (Republic of China)

## Abstract

**Background:**

Divorced women in Taiwanese and Chinese cultures face significant social stigma, yet research on their experiences remains limited. Applying the Gendered Life Course Perspective, this study examines how divorced older women’s marital transitions shape their identity reconstruction and how they establish new social roles beyond wifehood.

**Methods:**

This qualitative study used semi-structured interviews with 12 divorced older women from diverse backgrounds. Thematic analysis identified patterns in identity reconstruction and role transitions post-divorce.

**Findings:**

Divorced older women’s role transitions and identity reconstruction reveal distinctive patterns:1.Traditional roles and marital adversity: Participants endured unequal labor division, extensive caregiving, and significant conflicts, including infidelity and domestic violence. Many prioritized family stability over personal wellbeing, reflecting internalized gender expectations that defined their identities through wifehood and motherhood. 2. Identity disruption and reconstruction: Post-divorce, women faced economic vulnerability, emotional strain, health complications, and social stigma, forcing them to reconstruct their social identities beyond former roles as wives. 3. Community integration as identity reinforcement: Participants actively sought community involvement, social services, and employment opportunities to validate their reconstructed identities, creating spaces where their post-divorce identities could be affirmed outside traditional family structures.

**Conclusions and Implications:**

Divorced older women undergo significant role transitions that reshape their identities and aging experiences. Their post-divorce identity reconstruction highlights the need for targeted policies addressing economic vulnerability while creating community spaces that validate their new social roles. Interventions should include emotional support systems and reminiscence programs that help process past compromises, reducing stigma while promoting community integration.

